# Case report: Timing of eculizumab treatment in catastrophic antiphospholipid syndrome

**DOI:** 10.3389/fimmu.2024.1460317

**Published:** 2024-09-10

**Authors:** Camillo Carrara, Blerina Mataj, Sara Gastoldi, Piero Ruggenenti, Savino Sciascia, Dario Roccatello

**Affiliations:** ^1^ Unit of Nephrology, Azienda Socio-Sanitaria Territoriale Papa Giovanni XXIII, Bergamo, Italy; ^2^ Istituto di Ricerche Farmacologiche Mario Negri Istituto di Ricovero e Cura a Carattere Scientifico (IRCCS), Bergamo, Italy; ^3^ University Center of Excellence on Nephrologic, Rheumatologic and Rare Diseases (ERK-Net, ERN-Reconnect and RITA-ERN Member) with Nephrology and Dialysis Unit and Center of Immuno-Rheumatology and Rare Diseases (CMID), Coordinating Center of the Interregional Network for Rare Diseases of Piedmont and Aosta Valley, San Giovanni Bosco Hub Hospital ASL Città di Torino and Department of Clinical and Biological Sciences, Turin, Italy

**Keywords:** antiphospholipid syndrome, complement system, eculizumab, kidney failure, timing, catastrophic antiphospholipid antibody syndrome (CAPS), C5b-9

## Abstract

Catastrophic antiphospholipid syndrome (CAPS) is a life-threatening condition of small-vessel thrombosis with acute multiple-organ involvement and visceral damage. In this report, we present a case of a patient with CAPS who is refractory to conventional therapy. For the first time in a patient with CAPS, marked C5b-9 formation was demonstrated on microvascular endothelial cells, suggesting the usefulness of therapeutic complement inhibition in this setting. Eculizumab, a C5-blocking monoclonal antibody, is remarkably effective in the treatment of different forms of thrombotic microangiopathy by controlling complement system hyperactivation. It halted the “thrombotic storm” and promptly achieved full recovery of thrombocytopenia. However, kidney function did not recover, possibly because eculizumab was administered too late. Conceivably, the timing of treatment is crucial to achieving disease remission before irreversible structural damage occurs in target organs, thereby preventing their complete functional recovery.

## Introduction

The antiphospholipid syndrome (APS) is the most common cause of acquired immune-mediated thrombophilia (1). APS may present with a variety of clinical phenotypes, including thrombosis in the veins and arteries, as well as obstetrical morbidity (2). The pathogenesis of APS is not yet completely elucidated; however, pathways involving several cells (including platelets, leucocytes, and endothelial cells), as well as activation of the coagulation and complement systems, are all known to be involved in the development of thrombosis (3). About 1% of APS patients (4) develop the highly thrombotic phenotype referred to as catastrophic APS (CAPS). CAPS is a life-threatening variant of APS, with a mortality rate of approximately 30%, that is characterized by multiple organ involvement in a short period of time, mainly due to small vessel occlusions, often showing refractoriness to standard treatment with anticoagulants, glucocorticoids, and plasma exchanges (PEX) (5). Evidence shows that uncontrolled complement activation plays a central role in the pathogenesis of APS, particularly in CAPS (6). The hypothesis of a crucial involvement of activated complement in the pathogenesis of the disease is corroborated by evidence that in affected patients, as observed for patients with atypical hemolytic uremic syndrome (aHUS), there is a high prevalence of complement regulatory gene mutations (7). Based on the observation that C5 complement inhibition radically improved outcomes in patients with aHUS, eculizumab has been successfully administered as a prophylactic treatment in a CAPS patient undergoing renal transplantation (8–10). Following this preliminary experience, there have been an increasing number of clinical reports describing the benefits of eculizumab therapy in patients with CAPS that have been recently collected in the “CAPS registry” (11, 12).

However, despite effective complement inhibition (13) and resolution of thrombocytopenia, as well as the life-threatening “thrombotic storm” in patients refractory to conventional therapy, eculizumab may occasionally fail to achieve functional recovery in target organs, including the kidney. The clinical outcome of the patient we are describing in the present report suggests that optimal timing of eculizumab treatment is crucial to achieving disease remission before irreversible structural damage occurs in target organs, potentially preventing their complete functional recovery.

## Case report

We report the case of a 45-year-old man with a past medical history of triple-positive primary APS, characterized by a previous episode of pulmonary thromboembolism (PTE) and lower limb ulcers, who was on Warfarin and had a caval filter placed. He was also previously diagnosed with heparin-induced thrombocytopenia.

On 4 October 2022, he presented to our Emergency Department with intense asthenia and dyspnea. The arterial blood pressure was within the normal limits. His height and weight were, respectively, 176 cm and 76 kg. Laboratory tests showed severe pancytopenia (white blood cells: 2,760/μL, hemoglobin: 4.6 g/dL, platelets: 15,000/μL) along with B12 and folate deficiency. The computed tomography scan (CT) excluded a PTE and revealed numerous density alterations in the spleen parenchyma consistent with ischemic lesions. The patient received transfusion, and vitamin-K antagonist was discontinued, with the indication to start fondaparinux when the platelet count becomes higher than 20,000–30,000/μL.

**Figure 1:**
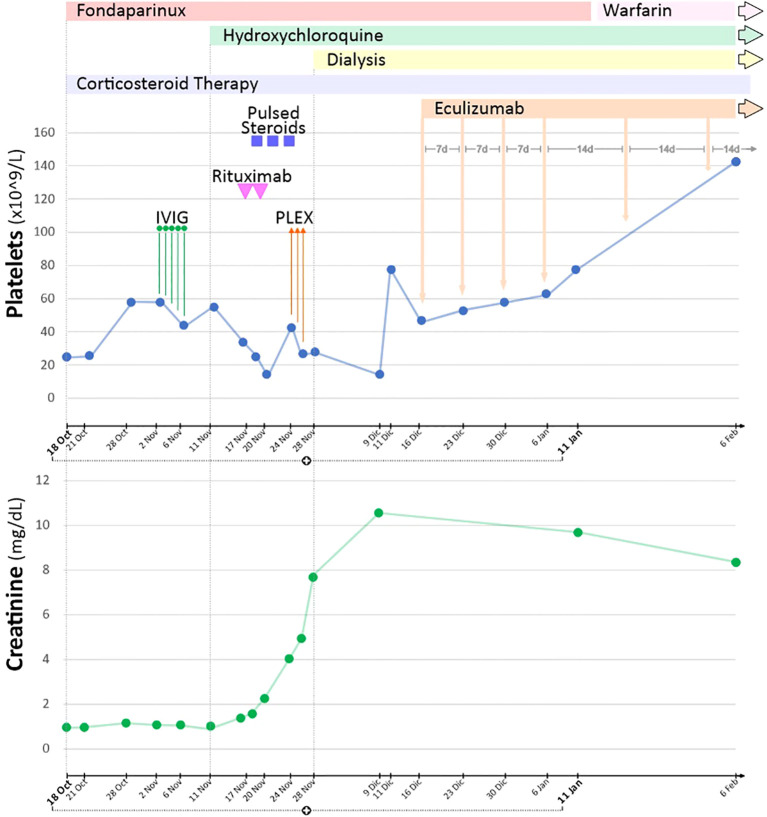
Patient treatment and outcome from admission until hospital discharge and follow-up.

**Figure 2:**
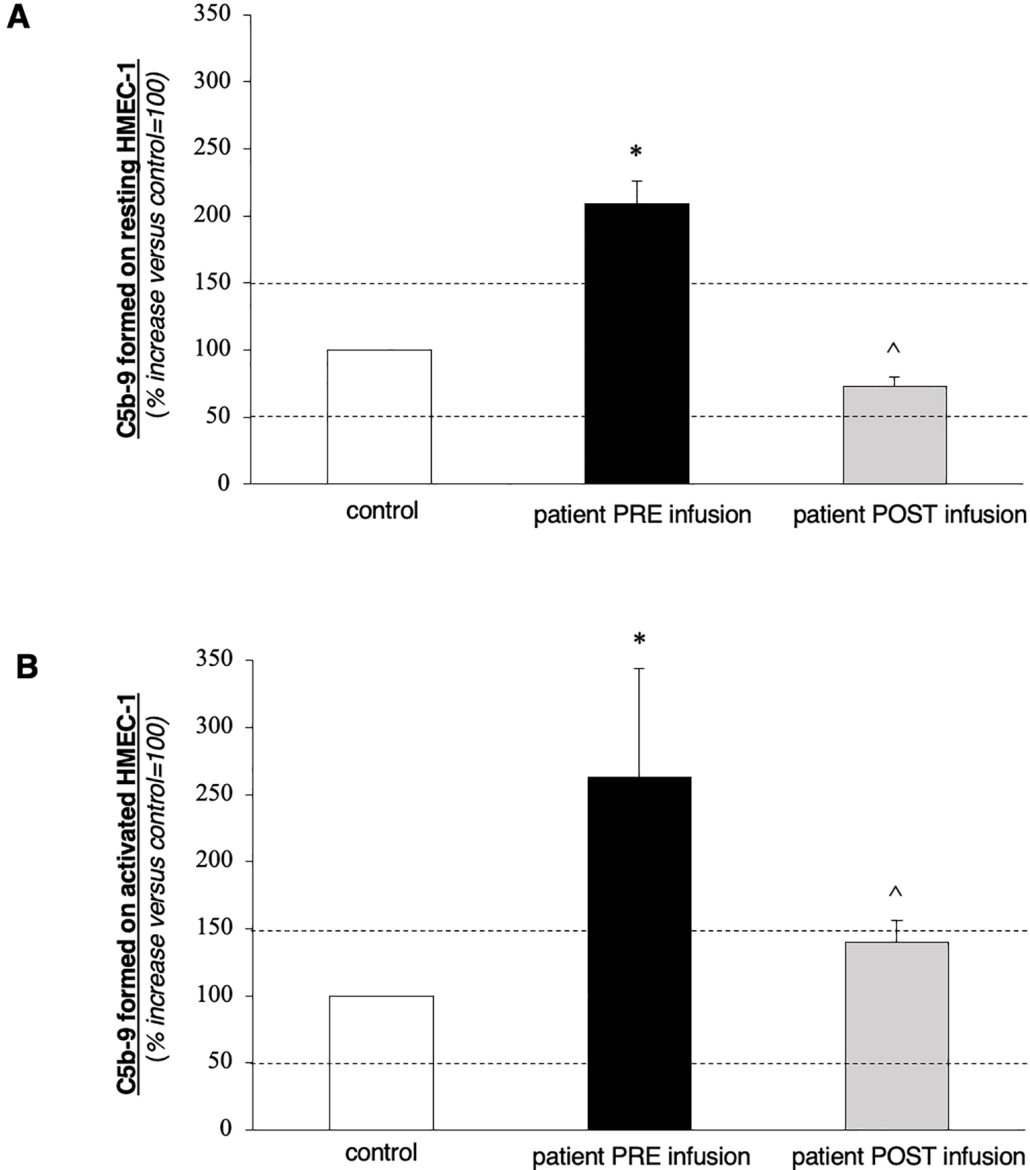
*Ex vivo* C5b-9 deposition on cultured human microvascular endothelial cell (HMEC-1) line induced by serum of the patient collected before (PRE-infusion) and 10 days after (POST-infusion) eculizumab treatment. **(A, B)** HMEC-1 resting **(A)** or activated with ADP **(B)** were incubated for 2 h with serum (diluted 1:2 with test medium, Hank’s Balanced Salt Solution (HBSS) with 0.5% Bovine Serum Albumin (BSA)) from the patient or with a control serum pool. At the end of incubation, cells were washed, fixed, and stained with rabbit antihuman complement C5b-9 complex antibody followed by Fluorescein Isothiocyanate (FITC)-conjugated secondary antibody. Fluorescence microscopy was used to view the fluorescent staining on the endothelial cell surface, and the HMEC-1 area covered by C5b-9 staining was calculated by automatic edge detection (Image J software) in 15 high-power fields. For each sample, the highest and lowest values were discarded, and the mean of the other 13 fields was calculated. Values were expressed as the percentage of C5b-9 deposits induced by a pool of sera from 10 healthy controls run in parallel (reference, 100%). Dashed lines indicate the upper and lower limits of the normal range. Data are reported ± SE. ^*^ *p* < 0.0001 versus control serum pool; °*p* < 0.0001 versus patient PRE-infusion. Statistical analysis: ANOVA.

After discharge from the emergency department, further laboratory examinations highlighted a polyglandular autoimmune syndrome, characterized by atrophic gastritis with anti-gastric parietal cells antibodies and autoimmune hypothyroidism. Pancytopenia at that time was considered to be based on vitamin deficiency, so a supplementing treatment with vitamin B12 and folate was started. Due to the improvement of thrombocytopenia (platelets up to 40,000/μL), fondaparinux was soon initiated. After 10 days, he was admitted again to our hospital, this time with the main complaint of abdominal pain and fever. The CT scans revealed acute acalculous cholecystitis and an extension of the splenic infarctions. Initial laboratory tests (**Table 1**) revealed thrombocytopenia and increased C-reactive protein (CRP) level. Empiric antibiotic therapy with piperacillin/tazobactam was started, along with hydration and parenteral nutrition, which resulted in mild clinical improvement and an initial CRP reduction. During hospitalization, he started complaining of severe headaches. Brain CT scans together with magnetic resonance imaging, were performed, which showed small ischemic lesions in different cortical areas and in the white matter. Considering the association of APS with severe thrombocytopenia and multiple organ infarcts, a diagnosis of CAPS was made. Fondaparinux dosage was increased, and corticosteroid treatment (3 days of intravenous methylprednisolone at the doses of 1 g + 0.5 g + 0.5 g, continuing thereafter with prednisone at 1 mg/kg/day, with subsequent tapering) was started along with hydroxychloroquine. Workup for autoimmune disorders ruled out systemic lupus erythematosus (SLE) (**Table 1**), and ADAMTS13 activity was found to be normal, with no ADAMTS13 inhibitor autoantibodies identified. Schistocytes were negative throughout the entire disease course. Notably, there was a marked C4 hypocomplementemia, together with low C3, while haptoglobin was consumed in only one determination. Due to persistent thrombocytopenia, on 2 November, he received a 5-day course of intravenous immunoglobulins (2 g/kg total dose), with no clinical or biochemical improvement (**Figure 1**). Therefore, rituximab therapy was added (two doses of 375 mg/m^2^ administered a week apart). Simultaneously, he developed a rapidly progressive renal failure with an active urinary sediment (microhematuria and proteinuria of 0.8 g/24 h) and arterial hypertension. Renal biopsy was considered too risky due to ongoing anticoagulation and severe thrombocytopenia, especially since the expected histological finding, given the prolonged anuria and tissue ischemia, would likely have been diffuse cortical necrosis. On 18 November, the patient was started on pulsed methylprednisolone (1 g + 0.5 g + 0.5 g), with subsequent tapering and three sessions of PEX. Unfortunately, kidney failure progressed, and hemodialysis treatment was started on 28 November. The patient did not show clinical improvement and continued to experience abdominal pain and thrombocytopenia, despite PEX and steroids. Marked C5b-9 formation was found on the microvascular endothelial cells (208% on resting and 263% on activated endothelial cells, normal values < 150%) (**Figure 2**), which completely normalized after exposure to eculizumab *in vitro* (13). The assessment of complement regulatory genes through NGS revealed a polymorphic heterozygous deletion of the CFHR3/CFHR1 genes.

**Table 1:** Laboratory values of the patient at baseline.

Variable	Value	Normal values
White cell count (per μL)	7,140	2,000–6,700
Red cell count (per μL)	3,040	4,700–5,820
Hemoglobin (g/dL)	9.8	14–17
Hematocrit (%)	28.5	43.1–51.5
Platelet count (per μL)	25,000	150,000–400,000
Creatinine (mg/dL)	0.99	0.30–1.30
Urea nitrogen (mg/dL)	23	10–50
Sodium (mmol/L)	140	136–145
Potassium (mmol/L)	3.9	3.50–5.00
Total bilirubin (mg/dL)	1.3	0.2–1.2
Aspartate aminotransferase (U/L)	30	13–40
Alanine aminotransferase (U/L)	23	7–40
d-Dimer (ng/mL)	2,282	< 500
Prothrombin time (PT) (ratio)	1.81	0.80–1.25
Activated partial thromboplastin time (ratio)	2.51	0.80–1.25
Lactate dehydrogenase (U/L)	842	120–246
Haptoglobin (g/L)	1.11	0.40–2.80
Schistocytes	Negative	Negative
C-reactive protein (mg/dL)	6	< 1.0
C3 (mg/dL)	0.56	0.79–1.52
C4 (mg/dL)	0.03	0.16–0.38
Anti-ADAMTS13 IgG (U/mL)	7	< 12–15
ADAMTS13 activity (%)	16	50–150
Coombs’ test	Negative	Negative
Antinuclear antibodies (ANA)	Negative	< 1/160
Anti-DNA antibodies (U/mL)	12	Negative < 27
Extractable nuclear antigen antibodies (ENA)	Negative	Negative
Anti-tireoperossidase antibodies (U/mL)	3,847	< 60
Anti-tireoglobulin antibodies (U/mL)	38	< 4.5
Lupus anticoagulant	Positive	Negative
Anti-cardiolipin IgM antibodies (U/mL)	287.5	0.0–20.0
Anti-cardiolipin IgG antibodies (U/mL)	890.2	0.0–20.0
Anti-β2 glycoprotein IgM antibodies (U/mL)	477.8	0.0–20.0
Anti-β2 glycoprotein IgG antibodies (U/mL)	3,709.6	0.0–20.0

On 16 December, eculizumab was started (900 mg for 4 weeks, followed by 1,200 mg at week 5), and then continued every 2 weeks thereafter. The patient received the vaccinations recommended for meningococcal and pneumococcal diseases. In addition, due to concerns about profound immunosuppression, he also received oral cotrimoxazole for the prevention of *Pneumocystis carinii* pneumonia. On 9 January 2023, C5b-9 formation on the microvascular endothelial cells was found to be within the normal range (72% on resting and 140% on activated endothelial cells). The patient was discharged with improving thrombocytopenia (platelet count of 78 × 10^9^/L) but was still on hemodialysis, receiving eculizumab every 2 weeks. Anticoagulation therapy was shifted back to a vitamin-K antagonist. At 2 months follow-up, his platelet count kept increasing (127,000/μL), but he remained anuric.

## Discussion

Here, we present the case of a patient affected by APS who developed multiorgan thrombosis, severe thrombocytopenia, and dialysis-dependent kidney failure in the context of CAPS. The patient was treated with anticoagulants, steroids, intravenous immunoglobulins, PEX, and rituximab without benefit. Eventually, eculizumab administration led to the rapid normalization of platelet count and halted the “thrombotic storm”. However, kidney function did not recover, and the patient remained dialysis-dependent. During the active phase of the disease, we were able to demonstrate higher-than-normal C5b-9 deposit formation on the microvascular endothelial cells, which completely normalized after treatment with the C5-blocker. Genetic analysis revealed a polymorphic heterozygous deletion of CFHR3/CFHR1 genes. Thus, we found a minor genetic abnormality in the complement system that could have predisposed to the development of CAPS upon exposure to a non-identified potential trigger.

CAPS is the most severe and rare form of APS, characterized by acute multiple-organ involvement, small-vessel thrombosis, and visceral damage (4). Complement has been identified as a critical pathway for the development of CAPS (14). Current knowledge supports the combination of high-dose glucocorticoids and the anticoagulant, heparin, as first-line treatment, with the addition of PEX and/or intravenous immunoglobulins for those with this life-threatening situation (5). Given the rarity and complexity of CAPS, as well as the absence of a clear consensus on the optimal timing of PEX initiation, it is challenging to determine whether earlier intervention would have significantly altered the final outcome in this case. Despite this standard treatment, CAPS is still associated with significant mortality. Several case reports suggest the efficacy of eculizumab in the management of CAPS (15–21). Furthermore, data from the “CAPS registry”, which includes 584 patients, of whom 6.7% were treated with eculizumab, suggest that complement inhibition should be considered in some patients with CAPS refractory to previous therapies, particularly if they present with features of aHUS (12). In the CAPS episode, 74.4% of patients treated with eculizumab recovered from the CAPS episode without recurrence of thrombosis during a median follow-up of 10.7 months. Among them, 64.1% presented complete remission and 10.3% had partial remission (improvement of hematologic features, but persisting signs of organ damage). All patients with thrombocytopenia showed an improvement in platelet count.

In a recent case series of patients with APS and thrombotic microangiopathy treated with eculizumab, an improvement in laboratory parameters was reported as early as the first week after the initiation of eculizumab. This suggests that complement inhibition might be considered earlier in the course of the disease (22). Timing in the administration of the anticomplement drug seems to be important in determining patient outcomes, especially the evolution of kidney function, as suggested in the “French cohort” (23). A total of 11 patients with severe CAPS and triple APS positivity were included in this study. Despite full doses of anticoagulants, corticosteroids, and PEX, the patients’ conditions continued to worsen. Overall, five patients had significant improvement in the few days after the first dose of eculizumab. Compared with nonresponders, responders had a less severe history of APS and a higher frequency of SLE. There was a higher number of patients who required hemodialysis before eculizumab in nonresponders. Among the four responders with renal failure, renal function recovered in three who were not on hemodialysis before eculizumab; the last patient continued to have dialysis-dependent renal failure. The authors concluded that patients with refractory CAPS respond inconsistently to eculizumab. Similar to our case, also in the French cohort, thrombocytopenia improved more rapidly and frequently after eculizumab, which may be of importance in sustaining optimal anticoagulation. The delay between the onset of CAPS and the administration of eculizumab raises the question of the benefit of earlier administration to avoid the occurrence of irreversible lesions, such as renal cortical necrosis that usually impairs renal function (24).

In conclusion, our case report demonstrates that inhibition of the complement system using eculizumab is a valuable strategy in the setting of CAPS. C5b-9 formation on cultured microvascular endothelial cells, a sophisticated test that we performed for the first time in a patient with CAPS, could represent a reliable marker of complement activation in the solid phase, and its inhibition is a reliable marker of the complement-inhibitory effect of eculizumab not only in aHUS but also in this setting. The timely administration of drugs (as early as possible) seems to be a crucial factor in preventing irreversible kidney structural damage and achieving full kidney function recovery.

## Data Availability

The original contributions presented in the study are included in the article/supplementary material. Further inquiries can be directed to the corresponding author.
